# Raddeanin A (RA) Inhibited EMT and Stemness in Glioblastoma via downregulating Skp2

**DOI:** 10.7150/jca.95266

**Published:** 2025-01-01

**Authors:** Weiqiang Jia, Yanling Zhang, Qiang Zhao, Min Gong, Yang Cao, Jia Liu, Shuang Luo

**Affiliations:** 1 Cancer Prevention and Treatment Institute of Chengdu, Department of Neurosurgery, Chengdu Fifth People's Hospital (The Second Clinical Medical College, Affiliated Fifth People's Hospital of Chengdu University of Traditional Chinese Medicine), Chengdu 611137, China.; 2 The First Clinical School of Guangzhou University of Chinese Medicine, Guangzhou 510000, China.; 3 Department of Oncology, The Forth Affiliated Hospital of Guangzhou Medical University, Guangzhou 510000, China.

**Keywords:** Raddeanin A, Glioblastoma, Skp2, EMT, GSCs

## Abstract

**Background:** Glioblastoma (GBM), notorious for its poor prognosis, stands as a formidable challenge within the central nervous system tumor category, primarily due to its intricate pathology that encompasses stemness and the epithelial-mesenchymal transition (EMT). The ubiquity of S phase kinase-associated protein 2 (Skp2) overexpression in GBM, a protein implicated in both EMT and stemness traits, correlates with increased drug resistance, elevated tumor grades, and adverse outcomes. This investigation delves into the impact of Raddeanin A (RA), a triterpenoid compound extracted from Anemone raddeana Regel, on GBM, with a special focus on its influence over Skp2 expression levels.

**Method:** The study assessed RA's influence on GBM cell lines U87 and U251 via CCK-8 and colony formation assays to gauge cell proliferation, alongside Transwell assays for evaluating migration and invasion capabilities. mRNA expression was detected by RT-PCR. Protein expression alterations were examined through western blotting and immunofluorescence techniques. The therapeutic potential of RA *in vivo* was also evaluated using subcutaneous and intracranial xenograft model in mice, developed using U87 cells. The molecular docking experiment was performed to evaluate the binding of RA to Skp2.

**Results:** RA markedly curtailed the proliferation of U87 and U251 cells in a concentration-dependent manner, alongside diminishing sphere formation in glioblastoma stem-like cells (GSCs). A significant suppression of Skp2 expression was observed in both cell lines and GSCs following RA treatment. This reduction in Skp2 was associated with a decrease in stemness markers (Sox2, Nestin) and the inhibition of EMT markers (Vimentin, N-cadherin, Snail). Moreover, Skp2 overexpression was found to mitigate RA's suppressive effects on EMT and stemness, highlighting Skp2's crucial role in these processes. The *in vivo* experiments supported these findings, indicating that RA not only thwarted tumor growth but also substantially lowered the expression of Skp2, EMT markers, and stemness indicators. Additionally, molecular docking experiments demonstrated that RA exhibits a notable binding affinity to Skp2.

**Conclusion:** This study elucidates RA's significant antitumor efficacy against GBM *in vitro* and *in vivo* by targeting pathways linked to stemness and EMT, chiefly via the downregulation of Skp2. These findings underscore RA's therapeutic promise in GBM management, offering insights into its mechanism of action and laying the groundwork for subsequent clinical investigations.

## Introduction

Glioblastoma (GBM) stands as one of the most formidable challenges in neuro-oncology, recognized as the most aggressive and malignant form of brain cancer. Characterized by rapid and invasive growth, GBM's clinical trajectory is marked by high morbidity and mortality. Despite significant advances in neurosurgery, radiation therapy, and chemotherapy, the prognosis for GBM patients remains bleak. Post-diagnosis survival rarely extends beyond months, reflecting the inadequacy of current treatment modalities. A particular point of concern is the inherent or acquired resistance to temozolomide (TMZ), the cornerstone of GBM chemotherapy, which significantly hampers treatment efficacy and patient survival.

The aggressive nature of GBM is further compounded by the process of epithelial-mesenchymal transition (EMT)^1^. EMT in cancer facilitates a transition from an epithelial phenotype to a mesenchymal state, endowing tumor cells with enhanced migratory and invasive capabilities. This process is foundational in the progression and metastasis of various cancers, including GBM. GBM cells undergoing EMT exhibit increased motility, invasiveness, and resistance to conventional therapies. Clinically, the activation of EMT in GBM correlates with a more malignant phenotype, marked by escalated cell migration, invasion, and poor patient outcomes. The presence of high levels of mesenchymal markers such as N-cadherin and Snail in GBM is associated with aggressive tumor behavior and a dismal prognosis^2^, ^3^. Targeting EMT, therefore, emerges as a promising therapeutic strategy to impede GBM progression and improve patient survival.

Concurrently, the role of cancer stem-like cells (CSCs), particularly glioma stem cells (GSCs) in GBM, has garnered significant attention. GSCs are implicated in tumor regeneration, chemotherapy resistance, and overall tumor maintenance^4^, ^5^. These cells exhibit the capacity for multi-lineage differentiation, self-renewal, and extensive proliferation, serving as crucial drivers of GBM growth and recurrence. The resistance of GSCs to standard chemotherapy and radiotherapy regimens presents a major hurdle in GBM treatment^6^, ^7^. Consequently, strategies aimed at targeting GSCs could potentially lead to more effective management of GBM, potentially inhibiting tumor growth and reducing recurrence rates.

In this complex landscape of GBM pathogenesis, the S phase kinase-related protein 2 (Skp2) emerges as a critical molecular player. Skp2, an oncogenic protein, is intimately linked to the resistance of tumors to drugs, the classification of tumor severity, and the outlook for patients^8^, ^9^. Its role has been characterized in several types of cancer, including GBM, prostate, breast, and lung cancers^10^. In the context of GBM, studies have demonstrated that the degradation of Skp2 leads to decreased cell proliferation and tumorigenesis, while also increasing cell sensitivity to TMZ^11^, ^12^. These findings position Skp2 as a valuable target in the quest for targeted therapy against GBM. Conversely, overexpression of Skp2 has been linked to increased drug resistance in breast cancer and osteosarcoma, mediated through its association with EMT^8^, ^13^. Artificial depletion of Skp2 has been shown to partially reverse the EMT phenotype and drug resistance, highlighting its potential as a therapeutic target. This relationship is also evident in GBM, where studies have shown that overexpression of Skp2 promotes the EMT phenotype, suggesting its significant role in tumor progression and treatment resistance.

Raddeanin A (RA), an oleanane-type triterpenoid saponin sourced from Anemone raddeana Regel, has garnered attention as a noteworthy compound. RA demonstrates anti-cancer effects across multiple cancer varieties, impacting essential signaling pathways vital for the survival and growth of cancer cells^14^-^16^. However, the specific effects and mechanisms of RA in GBM, particularly regarding its impact on Skp2-mediated EMT and stemness, have not been thoroughly explored. This research endeavors to fill this void by examining RA's suppressive impacts on Skp2-facilitated EMT and stemness within GBM. We hypothesize that RA could modulate Skp2 expression, thereby influencing the EMT process and the maintenance of GSCs. Through a detailed array of *in vitro* and *in vivo* tests, our study aims to shed light on RA's capability as an innovative therapeutic option for GBM. Through our investigation, we aim to contribute to the understanding of GBM pathophysiology, uncovering new therapeutic targets and mechanisms. This research could pave the way for the development of more effective, targeted therapeutic strategies against this formidable disease, offering hope for improved patient outcomes in the face of GBM.

## Materials and Methods

### Materials, reagents, and antibodies

Raddeanin A (RA), with a purity above 99% in its monohydrate version, was sourced from Tianjin Silan Technology Co., LTD, and kept at a temperature of 4℃. Both fetal bovine serum (FBS) and Dulbecco's modified Eagle's medium (DMEM) were purchased from Gibco, located in Grand Island, USA. For the purpose of immunodetection, Abcam (Cambridge, MA) provided antibodies for Snail (catalog No. ab180714) and N-cadherin (catalog No. ab76011). Meanwhile, Cell Signaling Technology (CST) in Beverly, MA, supplied the antibodies for Vimentin (catalog No. #5741), Skp2 (catalog No. #2652), Sox2(catalog No. #3579), Nestin (catalog No. #73349) and GAPDH (catalog No. #5174).

### Cell cultures

The U251 and U87 human glioblastoma cell lines, as well as the HA1800 and HEB normal glial cell lines, were obtained from the Chinese Academy of Medical Sciences (Beijing, China). These lines were grown in high-glucose DMEM enriched with 10% FBS and 1% penicillin/streptomycin. Dr. Shizhen Zhan from Sichuan University kindly provided the glioblastoma stem cells (GSCs) for this research. The GSCs were cultivated in a neurobasal medium containing 20 ng/ml of both bFGF and EGF from Peprotech, USA, along with 1% Glutamine and 2% B27 supplements from Gibco, USA.

### Cell viability assay

In the viability assay of cells, 3,000 cells were allocated per well in a 96-well plate and underwent a 24-hour incubation period. Subsequently, these cells were treated with varying concentrations of RA for another 24 hours. Cell viability was evaluated by adding 10 μl of the CCK-8 solution to each well, followed by an incubation period of one hour at 37°C. The optical density of each well was measured at a wavelength of 490 nm using a plate reader, facilitating a quantitative analysis of cell viability.

### Colony formation assay

The colony formation assessment involved creating single-cell suspensions, which were then plated at a rate of 400 cells per well. These cells were grown in a medium infused with different concentrations of RA, over a period ranging from 14 days. Following the incubation, the medium was removed, and cells were fixed using 4% paraformaldehyde for a duration of 10 minutes. Subsequently, the fixed cells underwent staining with crystal violet for an additional 10 minutes. After being washed, the colonies were counted to determine the impact of RA on the glioblastoma cells' ability to form colonies.

### Transwell assay

Migration and invasion tests were conducted using Transwell setups equipped with membranes having an 8-µm pore size (Corning, USA). In the migration test, 20,000 U87 and U251 cells were diluted in 200 µL of DMEM with 1% FBS and placed into the top chamber. The bottom chamber was filled with 600 µL of DMEM enriched with 20% FBS and various concentrations of Raddeanin A (RA). For the invasion experiments, the membranes in the top chambers were coated beforehand with 10 µL of Matrigel (BD Biosciences). Following a 24-hour period, the cells that had migrated or invaded through to the bottom chamber were fixed using ethanol, then stained with 0.2% crystal violet, and analyzed under a microscope across five randomly chosen fields at 100× magnification in three separate trials.

### Sphere formation assay

In the sphere formation assay, glioblastoma stem cells (GSCs) were seeded at a density of 2,000 cells per well in 6 cm ultra-low attachment plates (Corning) filled with GSC-specific medium. The medium was supplemented with varying levels of RA. Under standard growth conditions, after five days, spheres measuring over 50 µm in diameter were counted using a microscope. This procedure was repeated in triplicate, ensuring at least three visual fields were assessed for each replicate.

### Transfection

The U87, U251, and GSC lines underwent transfection with plasmids that either contained Skp2-flag or the flag tag alone (catalog No. F114966), sourced from Youbao Biotechnology Co., LTD, Changsha, employing the Lipofectamine 3000 agent as per the guidelines provided by the manufacturer. As a control, some cells were transfected with vacant vectors.

### Reverse transcription polymerase chain reaction (RT-PCR)

Total cellular RNA was extracted using Trizol reagent (Sigma-Aldrich). First-strand cDNA synthesis was performed using a PrimeScripTM RT reagent Kit, followed by PCR with Taq DNA polymerase (Takara, Dalian, China) and specific primers: human Skp2: 5'-AGGCTTAGATTCTGCAACTTG-3' (forward) and 5'-GCTGCTAAAGGTCTCTGGTGT-3' (reverse); human GAPDH: 5'-GGAGCGAGATCCCTCCAAAAT-3' (forward) and 5'-GGCTGTTGTCATACTTCTCATGG-3' (reverse).

### Western blotting

Protein extracts from xenograft glioblastoma tissues and cell lysates were processed using RIPA buffer (Guangzhou, China). Following SDS-PAGE separation on a 10% gel, proteins were transferred to PVDF membranes. These membranes were then blocked with 1× TBST containing 5% BSA at room temperature for one hour. Overnight at 4℃, membranes were incubated with primary antibodies diluted in 5% BSA (1×TBST), followed by incubation with secondary antibodies the subsequent day. The ECL chemiluminescence technique (Pierce, Rockford, IL, USA) was employed for protein visualization.

### Immunofluorescent staining

Glioblastoma Stem Cells (GSCs) were treated with 50 nM or 100 nM RA for five days. After treatment, the cells were placed on polylysine-coated glass slides for one hour. For immunofluorescence staining, the cells were first washed three times with PBS, followed by fixation with 4% paraformaldehyde in PBS for 10-15 minutes at room temperature. After fixation, the cells were washed three more times with PBS to remove any residual fixative. To permeabilize and block nonspecific binding, the cells were incubated with 0.3% Triton X-100 in goat serum (1:1000) for one hour at room temperature. This was followed by overnight incubation at 4°C with primary antibodies targeting Sox2 and Nestin, diluted 1:1000 in 5% BSA. The next day, after three PBS washes, the cells were incubated with the corresponding secondary antibodies for two hours at room temperature. Following the secondary antibody incubation, the cells were washed three more times with PBS, then stained with DAPI for 10 minutes to label the nuclei. After a final rinse, the samples were mounted using a resin-based mounting medium to preserve the fluorescence signals and imaged using a confocal microscope with light or fluorescence settings.

### RA treatment of a U87 subcutaneous xenograft model

Female BALB/c nude mice, aged 6-8 weeks and weighing 18-20g, were obtained from the Seventh Medical Center of the PLA General Hospital (Beijing, China), with all animal care protocols approved by the Institutional Animal Care Committee of the same institution, adhering to the People's Republic of China National Standard GB/T 35892 for laboratory animal welfare. For subcutaneous xenograft model, mice were dorsally inoculated with 5 × 10^6 U87 cells each. After five days, they were divided into two groups; one received RA intraperitoneal injections (100 mg/kg/day), and the other, vehicle injections. Tumor and body weights were monitored every five days. Upon conclusion, tumors were collected for analysis. For intracranial xenograft model, mice were intracranially injected with 1×10^6 U87 cells in 4 µL PBS (5 mice per group). The injections were performed using a Micro 4 Microsyringe Pump Controller connected to a Hamilton syringe equipped with a 33-gauge needle, targeting the mid-right striatum at specified coordinates from bregma: +0.5 anterior-posterior, +1.8 medio-lateral, -3.0 dorso-ventral. After three days, they were divided into two groups; one received RA intraperitoneal injections (200 mg/kg/day), and the other, vehicle injections. Following euthanasia, brains were harvested, and sliced coronally from the front to the back. Tumor size was determined by selecting the largest cross-sectional area of the tumor for measurement. Tumor volume was calculated using the formula V = (a × b^2) / 2, where 'a' represents the longest diameter and 'b' the shortest, with 'a' and 'b' being measured via Image pro plus. Survival analysis involved the intracranial injection of mice (five per group) following the procedure outlined above. Mice nearing death were euthanized under deep anesthesia, and the survivors were euthanized 40 days post-injection of U87 cells.

### Molecular docking

Molecular docking was performed with RA designed using ChemBio3D Ultra 14.0, starting from its structure obtained via PubChem based on its CAS number. Energy minimization was conducted, and the structure was saved in both "mol2" and "pdbqt" formats using AutoDockTools-1.5.6. The Skp2 protein was prepared by removing crystallization water and ligands in PyMOL 2.3.0, followed by charge calculations and atom typing with AutoDockTools, and saved in “pdbqt” format. Binding sites were predicted using POCASA 1.1, and docking was carried out with AutoDock Vina 1.1.2. The search space was set to a grid of 60*60*60 with a grid spacing of 0.375 Å and an exhaustiveness value of 10. Finally, the docking results were analyzed using PyMOL 2.3.0 to visualize and interpret the interaction between RA and the Skp2 protein.

### Statistical analysis

The mean ± standard error represents data aggregated from three replicates, analyzed using SPSS 21.0. An independent T-test was utilized for two-group comparisons, while a one-way ANOVA was employed for analyses involving multiple groups. Significance was assigned at a P-value below 0.05.

## Results

### RA inhibited the proliferation, migration, and invasion of GBM cells

**Figure 1:**
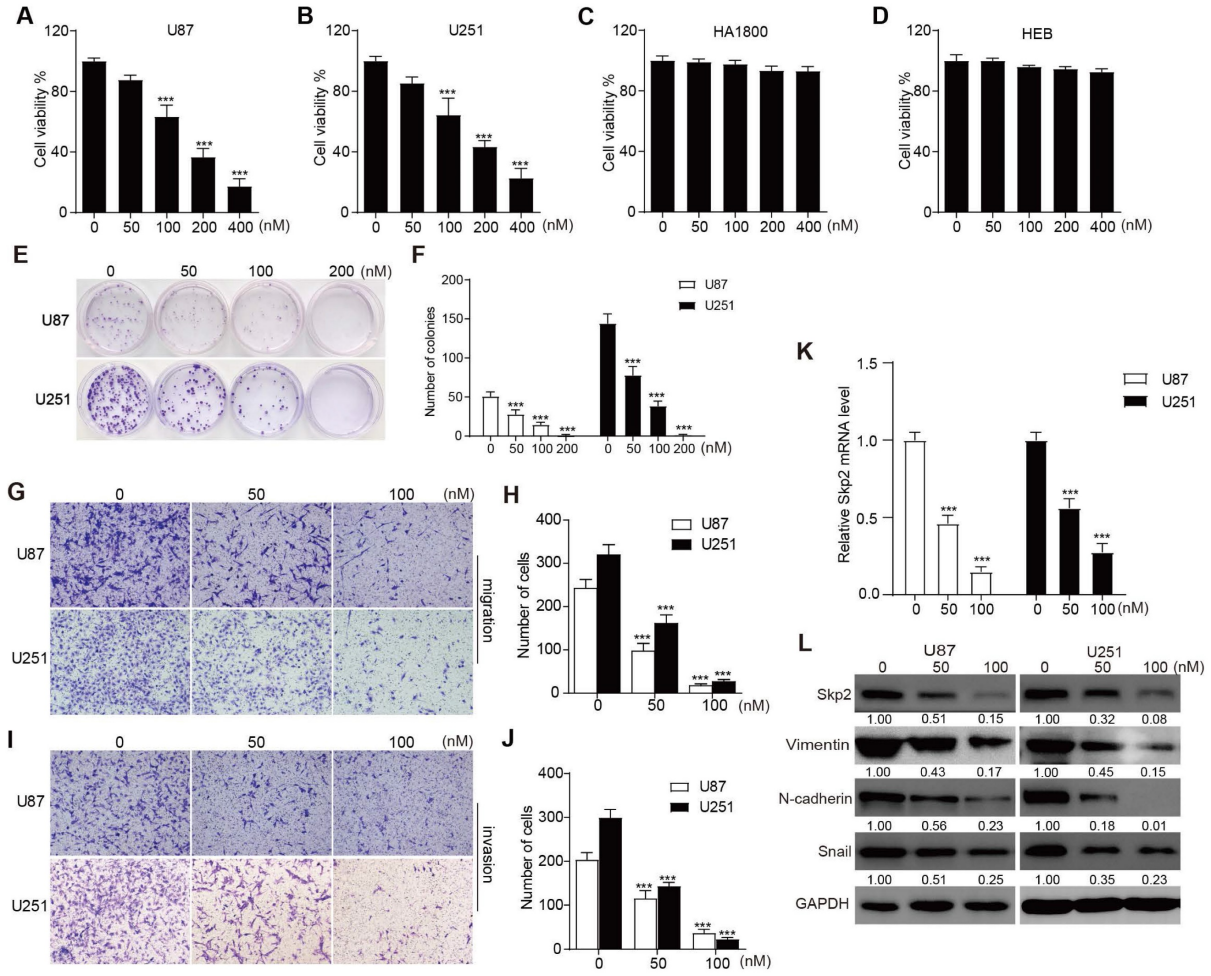
** RA inhibited proliferation, migration, invasion and decreases levels of Skp2 and EMT-related proteins in Glioblastoma Cells.** (A-D) The cell viability of U87, U251, HA1800 and HEB cells exposed to various concentrations of RA is depicted through CCK-8 assay outcomes. (E-F) The colony formation assay illustrates the proliferation potential of U251 and U87 cells upon treatment with specified doses of RA. (G-J) migration and invasion potential of U87 and U251 cells assessed by transwell assay after treatment with 50 nM or 100 nM RA for 24 hours (Scale bar = 100μm). (K) Analysis of Skp2 related mRNA levels in U87 and U251 cells treated with RA for 24 hours by RT-PCR. (L) Analysis of Skp2 and EMT-related protein levels in U87 and U251 cells treated with RA for 24 hours via western blot. Data are presented as mean ± SD for n = 3 experiments. Statistical significance is indicated as *p < 0.05; **p < 0.01; ***p < 0.001 when compared with the control group.

In assessing the anti-proliferative effects of RA on U251 and U87 glioblastoma cells, as well as HA1800 and HEB normal glial lines, CCK-8 assays were conducted. These assays revealed that concentrations of RA starting from 50 nM began to show inhibition of cell growth in U87 and U251, however, even at concentrations up to 400 nM, no significant toxicity was observed in HA1800 and HEB cells (Figure 1A-D). Additionally, colony formation assays demonstrated a significant inhibitory effect of the indicated RA in U87 and U251 (Figure 1E-F). These indicated that RA suppressed cell proliferation in a concentration-dependent manner in U87 and U251, without causing adverse effects on normal glial cell lines. To evaluate the anti-invasive and anti-migratory effects of RA, transwell assays were performed with RA concentrations of 50 nM and 100 nM. The results showed that cells treated with RA exhibited reduced degrees of cell invasion and migration (Figure 1G-J). These *in vitro* findings support RA's role as an inhibitor of glioblastoma cell proliferation, invasion, and migration.

### RA suppressed Skp2 and EMT-related markers in GBM cells

RT-PCR was used to evaluate the mRNA levels of Skp2 after 24 hours treatment with the indicated doses of RA. And western blotting was conducted on U251 and U87 cells treated with 50 nM and 100 nM RA for 24 hours to evaluate its effects on the EMT process and on Skp2 expression. Key EMT markers, Vimentin, N-cadherin and Snail, were analyzed. The results indicated that in both glioblastoma cell lines, RA downregulated Skp2 in mRNA level and RA suppressed levels of Skp2 protein and EMT-associated proteins in a dose-dependent manner (Figure 1K-L). This suggests that RA targets Skp2 in its suppression of EMT in glioblastoma cells.

### RA inhibited cell growth and downregulated Skp2 expression and stemness markers in GSCs

**Figure 2:**
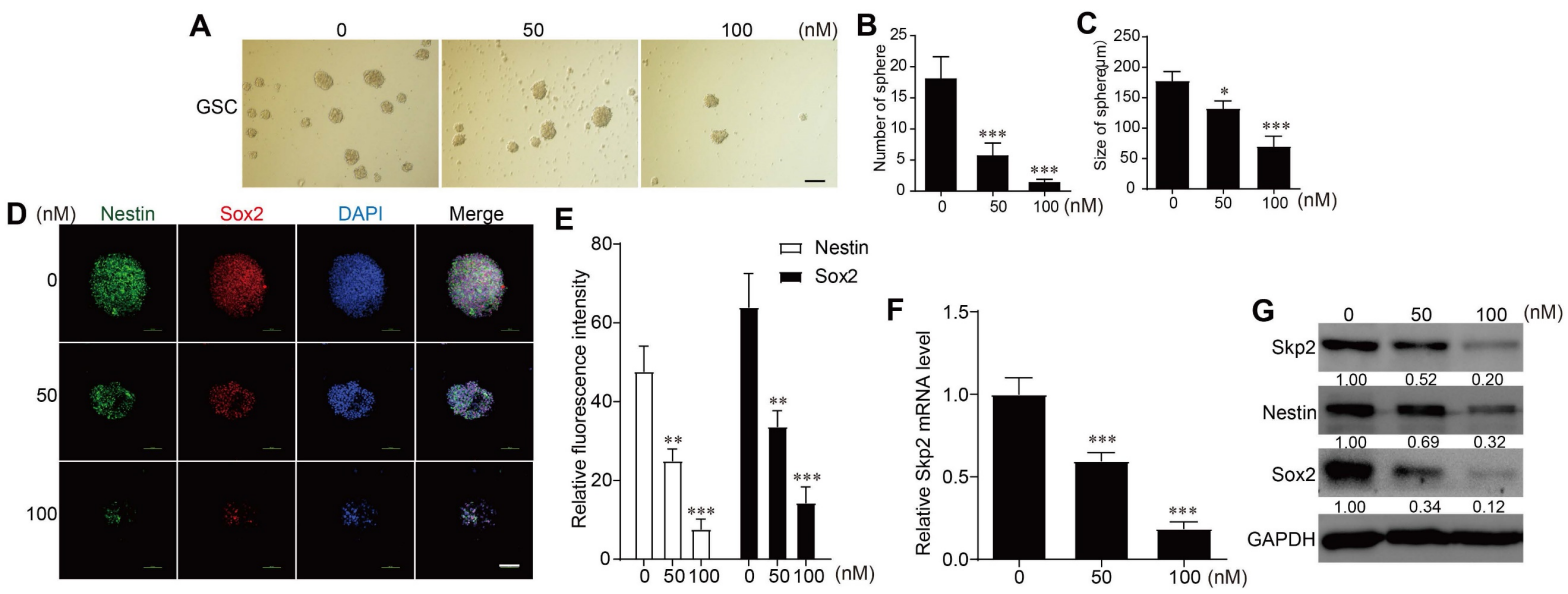
** RA Inhibited Cell Growth in Glioblastoma Stem Cells (GSCs) and Downregulates Skp2 and Stemness Markers.** (A-C) Assessment of sphere formation quantity and size to determine the effect of RA on GSCs at various doses (scale bar = 200μm). (D-E) Immunofluorescence assay for tracking expression of stemness proteins (Sox2 and Nestin) in GSCs (scale bar = 100μm). (F) Analysis of Skp2 related mRNA levels in GSCs treated with RA by RT-PCR (G) Quantification of Skp2, Sox2, and Nestin proteins in GSCs exposed to 50 nM or 100 nM RA over 5 Days, analyzed through western blotting. Data represent means ± SD for three independent experiments. Levels of statistical significance are marked as *p < 0.05; **p < 0.01; ***p < 0.001 when compared with the untreated control group.

In the sphere-forming assays with patient-derived GSCs, RA concentrations of 50 nM or 100 nM were used. Higher RA concentrations resulted in a reduced number and size of spheres formed, indicating that RA treatment suppressed GSCs sphere formation (Figure 2A-C). RA downregulated Skp2 in mRNA level in GSCs (Figure 2C). And immunofluorescence experiments to detect Nestin and Sox2 protein levels showed that these stemness markers decreased with increasing RA concentrations (Figure 2D-E). RT-PCR further confirmed that RA reduced Skp2 mRNA levels (Figure 2F). And western blot analysis further confirmed that RA decreased Skp2 protein levels and downregulated stemness marker expressions in a dose-dependent manner in GSCs (Figure 2G).

### Skp2 overexpression reduced RA effects on glioblastoma cells

**Figure 3:**
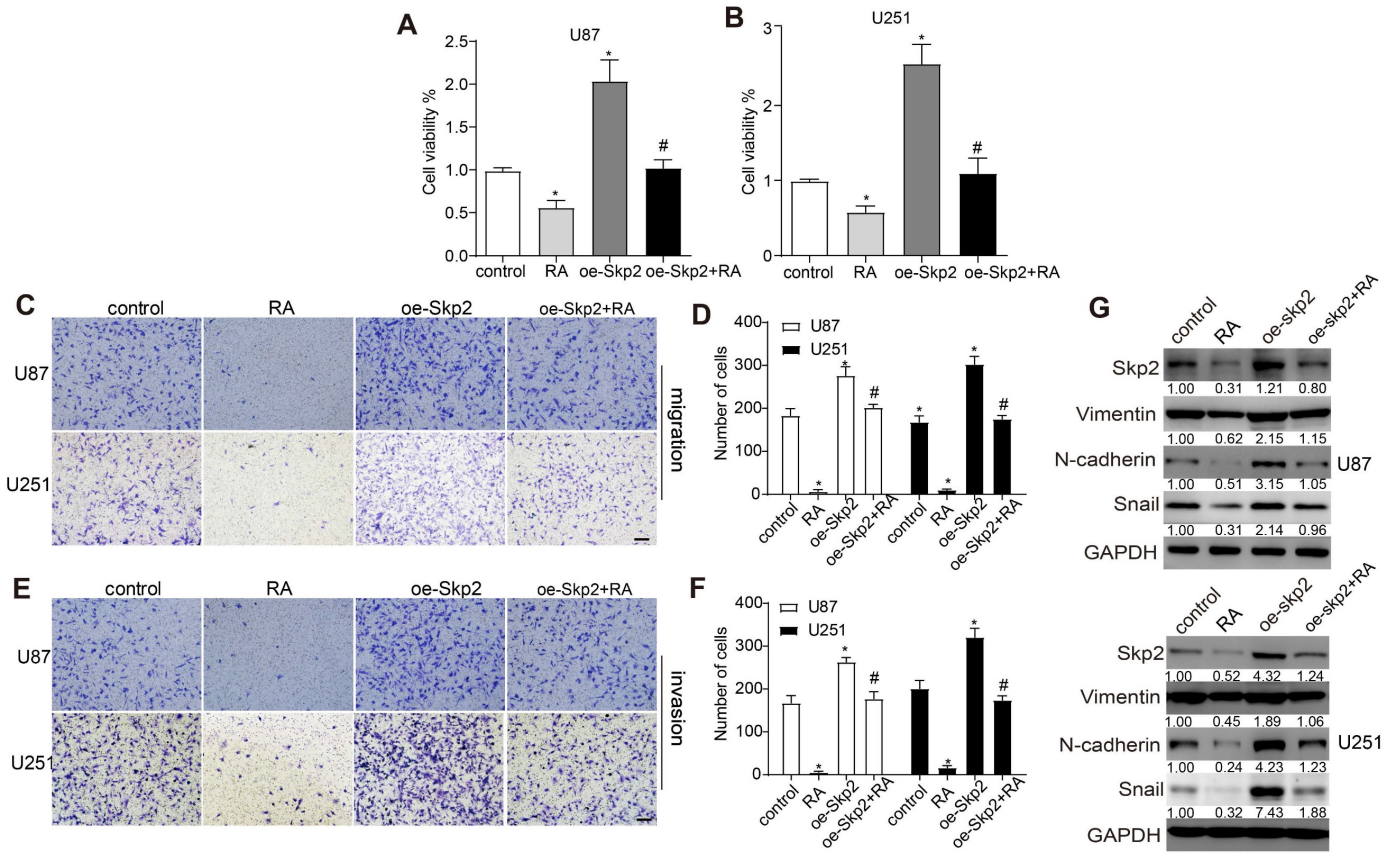
** Overexpression of Skp2 Diminishes the Efficacy of RA in GBM Cells.** Skp2 was artificially upregulated in U87 and U251 cells. (A-B) Cell survival rates were assessed through CCK8 assays. (C-F) The capability of cells to migrate and invade following RA treatment was evaluated via transwell assays (Scale bar = 100 μm). (G) Quantification of Skp2, N-cadherin, and Snail protein concentrations post-RA exposure was performed using Western blot techniques. Data are shown as mean ± SD for three replicates, with *p < 0.05 indicating statistical significance compared to the control group, and #p < 0.05 relative to the RA-treated cohort.

To elucidate Skp2's contribution to the effectiveness of RA, U251 and U87 cell lines were genetically engineered to augment Skp2 expression. This elevation in Skp2 levels resulted in increased cell proliferation, migration, invasion, and the upregulation of EMT markers such as N-cadherin and Snail. Notably, the augmentation of Skp2 expression diminished RA's suppressive impact on the invasion, migration, proliferation of glioblastoma cells, and the expression of EMT indicators RT-PCR further confirmed that RA reduced Skp2 mRNA levels (Figure 3). These observations indicate that RA's anticancer activities against glioblastoma might be partially attributed to its capacity to reduce Skp2-induced EMT enhancements.

### Skp2 overexpression decreased the effect of RA in GSCs

**Figure 4:**
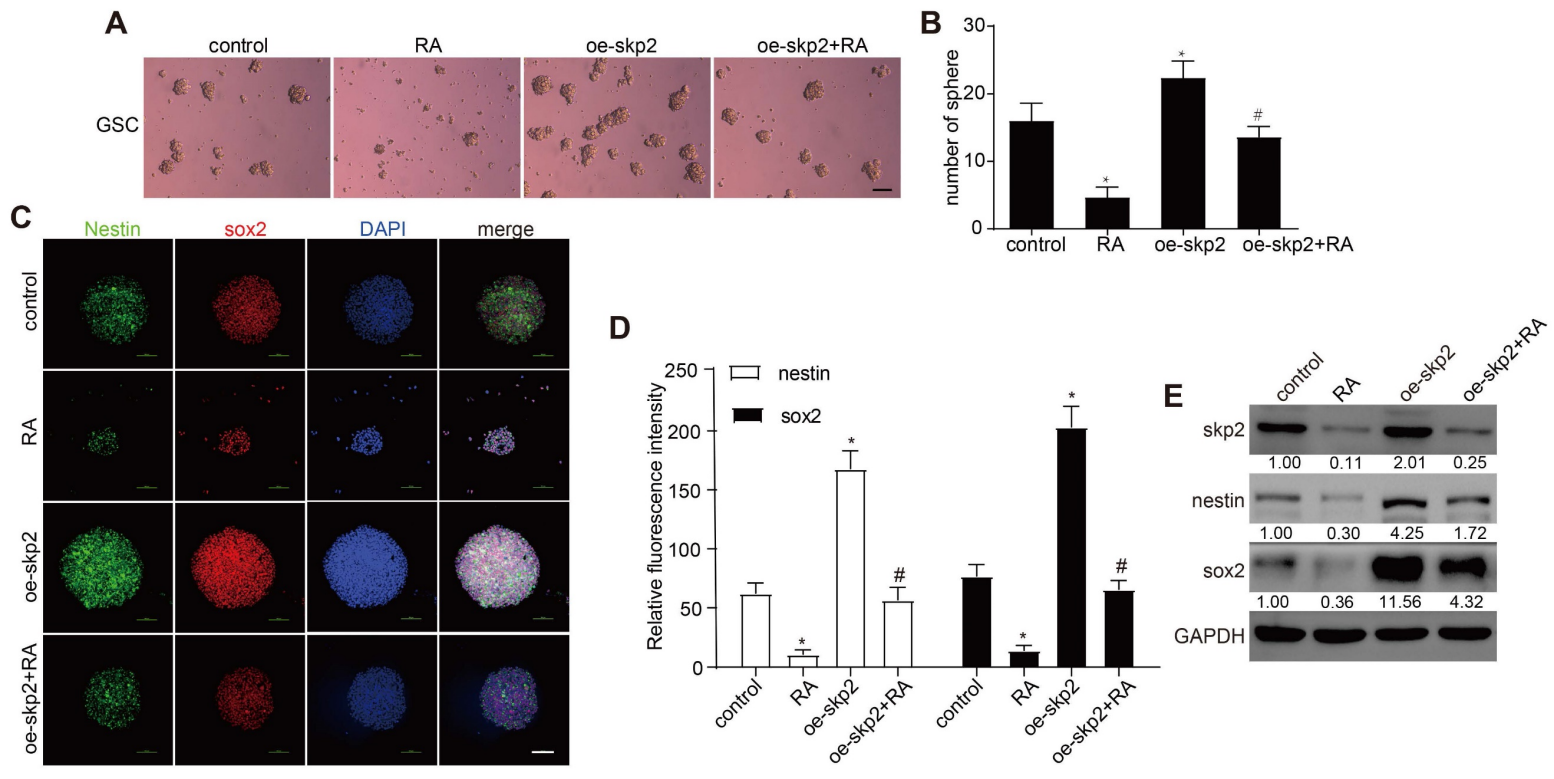
** Skp2 overexpression decreased the effect of RA in GSCs.** GSCs underwent transfection with either empty vectors or vectors promoting Skp2 overexpression, followed by treatment with 100 nM RA or no treatment. (A-B) Sphere formation assays were utilized to assess RA's impact on the proliferation of GSCs (Scale bar = 100μm). (C-D) The expression levels of stemness-associated proteins (Sox2 and Nestin) were evaluated through immunofluorescence techniques (Scale bar = 100μm). (E) Western blot analysis was conducted to measure the levels of Skp2 protein and stemness indicators in GSCs. Statistical data are presented as mean ± SD for three experiments, with *p < 0.05 indicating significance compared to the control group, and #p < 0.05 indicating significance compared to the RA-treated group.

Subsequent investigations assessed whether RA's ability to diminish stemness markers in GSCs was contingent upon the inhibition of Skp2. GSCs, transfected to overexpress Skp2, were exposed to 100 nM RA, which revealed that Skp2 elevation increased sphere formation and boosted Nestin and Sox2 levels. Furthermore, the overexpression of Skp2 negated RA's impacts on sphere formation and the levels of stemness markers in GSCs RT-PCR further confirmed that RA reduced Skp2 mRNA levels (Figure 4). This evidence suggests that the anticancer effects of RA in GSCs may be ascribed to its anti-stemness properties, facilitated through the curtailment of Skp2.

### RA suppressed GBM growth in U87 xenograft mouse model

**Figure 5:**
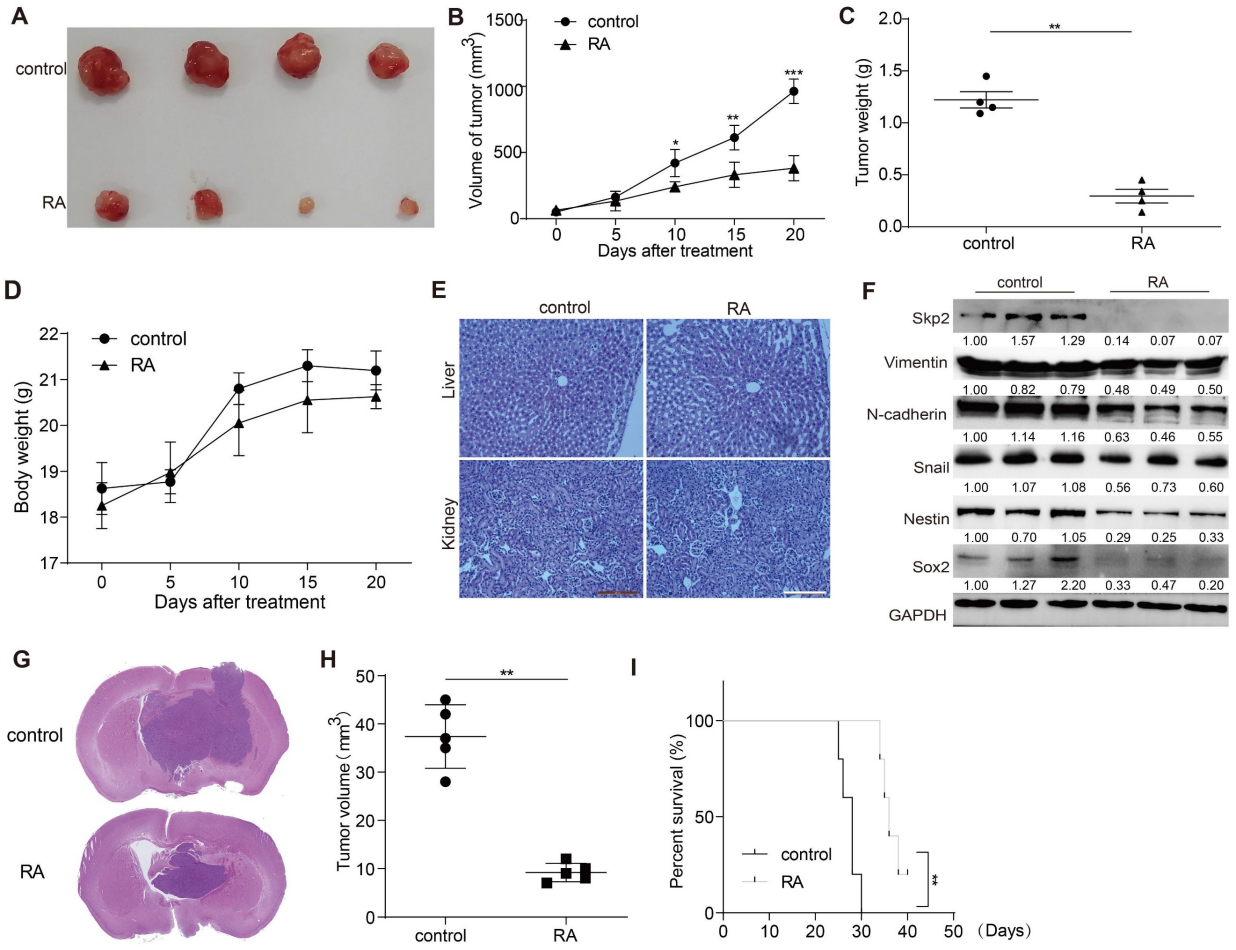
** RA decreases glioma growth in a xenograft mouse model.** (A) Visual documentation showcases the reduction in U87 xenograft tumor sizes. (B) Tumor volumes, (C) tumor weights, and (D) the body weights of mice were monitored and recorded at five-day intervals. (E) HE staining of liver and kidney sections from control and RA-treated mice. (F) At the end of the study, tumors were collected for protein expression analysis using Western blot. (G) Histological analysis of orthotopic gliomas in mice from the control and RA groups is shown. (H) Tumor sizes from orthotopic glioma xenografts were quantitatively assessed. (I) Kaplan-Meier survival curves for mice with glioma demonstrate a significant improvement in survival rates in the RA-treated group. Each experimental group included either three or four subjects. Repetitions of all experimental procedures were done three times. Significance levels are denoted as *P < 0.05, **P < 0.01, and ***P < 0.001 when compared to the control group.

The *in vivo* effectiveness of RA against GBM was appraised using a U87 xenograft mouse model. RA administration markedly hindered tumor expansion over a 20-day span compared to the vehicle-treated control group. The RA treatment group exhibited significant reductions in tumor size and mass without affecting the overall body weight (Figure 5A-D). Additionally, no abnormalities were observed in the liver and kidney tissue sections of the treated mice (Figure 5E). Aligning with *in vitro* findings, the expression levels of Skp2, EMT markers (N-cadherin and Snail), and stemness indicators (Nestin and Sox2) were also reduced in the RA treatment group (Figure 5F). Subsequently, we used a mouse intracranial glioma model to demonstrate the inhibitory effect of RA on gliomas. RA inhibited the growth of gliomas while increasing the survival time of mice with gliomas (Figure 5G-I). Collectively, these findings corroborate that RA impedes tumor progression *in vivo*.

### Interaction between RA and Skp2

**Figure 6:**
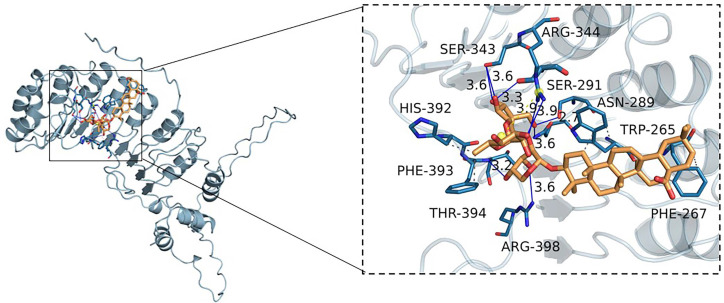
** RA's interaction with Skp2.** Molecular docking reveals RA's binding to the Skp2 active pocket with a binding energy of -8.9 kcal/mol. RA forms hydrogen bonds with residues SER343, ARG344, SER291, ASN289, and THR394, with bond lengths ranging from 2.7 to 3.9 Å. Hydrophobic interactions involve TRP265, PHE267, PHE393, and HIS392, stabilizing the ligand-protein complex. The close-up view shows the key interactions within the binding site.

To assess the potential interaction between RA and Skp2, we conducted molecular docking studies. The results revealed that RA exhibits a notable binding affinity for Skp2, characterized by a binding energy of -8.9 kcal/mol. By analyzing the three-dimensional interactions, the compound forms hydrogen bonds with the amino acid residues SER343, ARG344, SER291, ASN289, and THR394 of the protein. The hydrogen bond lengths are 3.6, 3.6, 3.3, 3.9, 2.7, 3.6, 3.6, and 3.2 Å, respectively. Additionally, the compound forms hydrophobic interactions with the amino acid residues TRP265, PHE267, PHE393, and HIS392 (Figure 6). These interactions facilitate the ligand's binding to the protein's active pocket, forming a complex.

## Discussion

Glioblastoma's rapid proliferation, aggressive migration, and high invasion propensity, contribute to its daunting prognosis and limited treatment options. The integration of Traditional Chinese Medicine (TCM) into modern oncological research has brought Raddeanin A (RA) into focus, particularly for its potential in treating glioblastoma. This study emphasizes RA's efficacy in targeting key biological processes involved in cancer progression. Our research specifically investigates RA's impact on glioblastoma cells, underscoring its value as a therapeutic agent. RA has emerged as a significant inhibitor of these malignancies in our studies, with pronounced suppression of cell proliferation, migration, and invasion via downregulating Skp2-indued EMT and stemness noted both *in vitro* and *in vivo*.

The epithelial-mesenchymal transition (EMT) process, critical in cancer metastasis and invasion, including in glioblastoma, has been a key focus. EMT entails the transformation of epithelial cells into a mesenchymal phenotype, granting them increased proliferation, motility, invasiveness, stemness and drug resistance^17^, ^18^. The EMT process in glioblastoma, though controversial, is supported by numerous experimental and clinical evidences. These studies demonstrate that an increase in mesenchymal markers is indeed associated with a poorer survival prognosis in glioblastoma patients. This relationship underscores the significance of EMT in understanding the progression and treatment responses of this aggressive brain tumor^19^. Targeting the EMT process in the treatment of glioblastoma has been a subject of numerous reports, and natural compounds play a significant role in this area. For instance, decorin inhibited invasion through EMT process suppression in glioma^20^. Galangin reduced proliferation, migration, and invasion in GBM cells by targeting EMT process^21^. However, there have been few reports on RA specifically targeting EMT to suppress glioblastoma. In previous study, Wu et al reported that RA inhibited EMT in glioblastoma by downregulating β-catenin expression^22^. In our study, we demonstrated RA inhibited migration and invasion via downregulation of Skp2-induced EMT, further confirming the efficacy of RA in targeting EMT for the treatment of glioblastoma. Glioma stem cells (GSCs), known to contribute to tumor recurrence and resistance to standard treatments, are found to enhance glioma malignancy when interacting with the EMT process^23^. Numerous studies have confirmed the existence of glioblastoma stem cells, and targeting glioblastoma stemness has become a key therapeutic strategy in treating this malignancy. The focus on glioblastoma stem cells stems from their role in tumor progression, resistance to conventional therapies, and recurrence^6^. Therapies that specifically target the unique characteristics of these stem cells offer a promising approach to improve treatment outcomes and potentially reduce the high recurrence rate associated with glioblastoma.

For example, a compound BRM270 from natural plant extracts inhibited glioblastoma stem cell properties and glioblastoma recurrence^24^. Alpinetin targeted glioma stem cells by suppressing Notch pathway^25^. In our study, we firstly validated RA's capability to attenuate stemness in glioblastoma stemness cells. This effect is marked by a decrease in stemness markers like Nestin and Sox2, attributed to the downregulation of Skp2, both *in vitro* and *in vivo*. This adds a new theoretical foundation for the application of RA in combating glioblastoma.

Skp2's role in EMT and stemness has been well-established. In breast cancer cells, Skp2 overexpression has been shown to boost proliferation and inhibit apoptosis, affecting cell cycle phases. Similar findings in lung cancer link increased Skp2 to enhanced metastasis and invasion via upregulation of MMP-2 and MMP-9^26^. In glioblastoma, elevated Skp2 expression correlates with poorer survival and prognosis. Studies suppressing Skp2 expression have led to decreased malignant cell proliferation and tumorigenesis, as well as increased sensitivity of glioblastoma cells to TMZ. In our study, our study marks the first report of RA's ability to downregulate Skp2. Our findings align with these observations, highlighting Skp2 as a promising target in glioblastoma treatment, with RA showing efficacy in reducing Skp2-mediated malignancy traits. However, whether Skp2 is a direct target of RA requires further experimental validation.

The efficacy of agents against GBM *in vivo*, especially their ability to cross the blood-brain barrier (BBB), continues to pose significant challenges. This study further explores the capability of RA to suppress glioma within a mouse stereotactic brain model, highlighting the importance of assessing the safety and effectiveness of potential drugs, especially those targeting cancer. Earlier studies conducted *in vivo* have confirmed the safety of RA, encompassing experiments on the central nervous system (CNS)^27^-^29^. In this investigation, RA successfully reduced GBM growth in a subcutaneous model and intracranial glioma model, aligning with our *in vitro* results, and exhibited tolerability without major side effects. Drug delivery to the brain, limited by the blood-brain barrier (BBB), remains a major hurdle in treating glioblastoma. Nanoparticle-based delivery systems offer a promising solution by improving drug bioavailability and targeting. By modifying nanoparticles to interact with receptors overexpressed on glioblastoma cells, such as transferrin, drug accumulation at the tumor site can be enhanced. Additionally, focused ultrasound (FUS) techniques can temporarily disrupt the BBB, allowing localized delivery of drugs, which could further improve the effectiveness of RA. These approaches, while promising, require further preclinical and clinical validation.

While this study provides promising insights into the anti-glioblastoma effects of Raddeanin A (RA), several limitations need to be acknowledged. First, although our findings suggest that RA may exert its effects through interaction with Skp2, direct experimental evidence of this interaction is lacking. Our conclusions are based on molecular docking simulations, which, while informative, do not substitute for empirical validation. Future studies using experimental techniques such as co-immunoprecipitation or surface plasmon resonance (SPR) will be essential to confirm the binding of RA to Skp2 at the protein level. Second, this study has not fully explored the underlying signaling pathways through which RA mediates its anti-glioblastoma effects. While RA's interaction with Skp2 hints at a potential mechanism, further research is required to delineate the specific signaling cascades involved. Identifying these pathways would offer a more comprehensive understanding of RA's role in glioblastoma treatment and help guide the development of targeted therapies. Future work should also focus on *in vivo* studies to evaluate the clinical relevance of these findings and to assess the efficacy of RA in combination with other therapeutic agents. Addressing these limitations will not only validate the proposed mechanisms but also help to optimize RA's potential as a therapeutic agent for glioblastoma.

To conclude, the thorough examination of Raddeanin A's impact on glioblastoma offers valuable perspectives on its viability as a cancer-fighting agent. Through the targeting of Skp2, RA impedes the proliferation, migration, and invasion of glioblastoma cells, and also proficiently counteracts EMT processes and reduces stemness attributes. These results lay the foundation for further research and clinical trials, aiming to establish RA as an effective therapeutic option for glioblastoma, potentially enhancing outcomes for patients facing this formidable disease.
